# 3D Texture Analysis Reveals Imperceptible MRI Textural Alterations in the Thalamus and Putamen in Progressive Myoclonic Epilepsy Type 1, EPM1

**DOI:** 10.1371/journal.pone.0069905

**Published:** 2013-07-29

**Authors:** Sanna Suoranta, Kirsi Holli-Helenius, Päivi Koskenkorva, Eini Niskanen, Mervi Könönen, Marja Äikiä, Hannu Eskola, Reetta Kälviäinen, Ritva Vanninen

**Affiliations:** 1 Department of Clinical Radiology, Kuopio University Hospital, Kuopio, Finland; 2 Medical Imaging Centre and Hospital Pharmacy, Tampere University Hospital, Tampere, Finland; 3 Department of Biomedical Engineering, Tampere University of Technology, Tampere, Finland; 4 Department of Clinical Neurophysiology, Kuopio University Hospital, Kuopio, Finland; 5 Department of Neurology, Kuopio University Hospital, Kuopio, Finland; 6 Institute of Clinical Medicine, Neurology, University of Eastern Finland, Kuopio, Finland; 7 Institute of Clinical Medicine, Clinical Radiology, University of Eastern Finland, Kuopio, Finland; Oregon Health & Science University, United States of America

## Abstract

Progressive myoclonic epilepsy type 1 (EPM1) is an autosomal recessively inherited neurodegenerative disorder characterized by young onset age, myoclonus and tonic-clonic epileptic seizures. At the time of diagnosis, the visual assessment of the brain MRI is usually normal, with no major changes found later. Therefore, we utilized texture analysis (TA) to characterize and classify the underlying properties of the affected brain tissue by means of 3D texture features. Sixteen genetically verified patients with EPM1 and 16 healthy controls were included in the study. TA was performed upon 3D volumes of interest that were placed bilaterally in the thalamus, amygdala, hippocampus, caudate nucleus and putamen. Compared to the healthy controls, EPM1 patients had significant textural differences especially in the thalamus and right putamen. The most significantly differing texture features included parameters that measure the complexity and heterogeneity of the tissue, such as the co-occurrence matrix-based entropy and angular second moment, and also the run-length matrix-based parameters of gray-level non-uniformity, short run emphasis and long run emphasis. This study demonstrates the usability of 3D TA for extracting additional information from MR images. Textural alterations which suggest complex, coarse and heterogeneous appearance were found bilaterally in the thalamus, supporting the previous literature on thalamic pathology in EPM1. The observed putamenal involvement is a novel finding. Our results encourage further studies on the clinical applications, feasibility, reproducibility and reliability of 3D TA.

## Introduction

Progressive myoclonic epilepsy type 1 or Unverricht-Lundborg disease (EPM1, ULD, OMIM 254800) is the most common type of progressive myoclonic epilepsy [1]. It is an autosomal recessively inherited neurodegenerative disorder caused by mutations in the cystatin B gene (*CSTB*) [2]–[4]. EPM1 in Finland has an incidence of 1∶20 000 births per year, with about 200 diagnosed cases [5]), but it is also prevalent elsewhere in the Baltic Sea region and in the Western Mediterranean area. Sporadic cases of EPM1 have been reported worldwide [6].

The first symptoms of EPM1 are commonly stimulus-sensitive myoclonic jerks and generalized tonic-clonic epileptic seizures. Neurological examination is initially normal, but patients later develop intention tremor, dysarthria, ataxia and poor coordination, thus subsequently one-third of EPM1 patients become severely incapacitated and wheelchair bound. Alternatively, the clinical symptoms can be so mild that there is a delay in the diagnosis and patients may manage well. [6] Mild cognitive impairment and slow decline in intellectual level over time have been reported [6]–[9].

MRI findings of the patients with EPM1 remain sparse. At the time of diagnosis, MRI of the brain is usually normal [6]. However, changes in MRI, such as mild to moderate cerebral and/or cerebellar atrophy, loss of neuronal volume in the brainstem and high intensity signal changes in the basal ganglia have been reported as well [7], [10]–[12]. Recently modern group level MRI analysis methods have revealed loss of gray matter volume in cortical motor areas (voxel-based morphometry, VBM), and atrophy of the sensorimotor, visual and auditory cortices (cortical thickness analysis, CTH) in EPM1 patients [13], [14] while abnormal findings could not be detected in visual assessments. Loss of gray matter volume in the thalamus has also been reported in one VBM study [13], paralleling a PET study indicating dopamine depletion in the thalamostriatal area in four EPM1 patients [15].

Although the human visual system can discriminate different textures, the capacity of human vision to detect and discriminate between complex higher-order textures is limited [16]. Texture analysis (TA) is a method to evaluate the position of signal features i.e. pixels/voxels, and their gray-level intensity, distribution and relationships in a digital image [17]. TA presents texture features as mathematical parameters, which could characterize the properties of the underlying tissue. These features can be described as, for example, fine, coarse, smooth, or irregular [18]. Previously, texture analysis techniques have been used two-dimensionally in medical imaging of multiple sclerosis, brain tumours and brain injuries [17], [19], [20]. In epilepsy research, TA has been applied in temporal lobe epilepsy, focal cortical dysplasia and juvenile myoclonic epilepsy (JME) [21]–[27].

Theoretically, three-dimensional (3D) texture analysis provides more comprehensive data analysis of biological tissue texture properties and enables calculation of texture parameters in several directions. However, the literature on 3D TA applications in brain research is still sparse [28]–[33].

Patients with EPM1 seem to provide a suitable population to assess the feasibility of novel image analysis techniques to detect possible subtle changes in the brain that are not evident upon visual assessment. The specific aim of this study is to investigate possible imperceptible structural differences in the thalamus and other deep gray matter tissue in patients with EPM1 via comparison with healthy controls by using three-dimensional MRI-based texture analysis. Further, we want to determine whether the possible texture changes correlate with EPM1 patients’ clinical symptoms and neuropsychological findings.

## Patients and Methods

### Subjects

EPM1 patients were evaluated at Kuopio University Hospital during the period 2006−2010. The study was jointly administered by the Folkhälsan Institute of Genetics and Neuroscience Center at the University of Helsinki. The patients had either participated in an earlier molecular genetics study or were referred to the Kuopio Epilepsy Center during the study. The ethics committee at the Kuopio University Hospital approved the study and written informed consent was obtained from all participants.

The original EPM1 study group comprised 66 EPM1 patients. In all cases, MR images were obtained using a T1-weighted 3D magnetization-prepared rapid acquisition of gradient echo sequence. Due to slight differences in updated scanner versions, technical difficulties, and slightly different slice thicknesses in some of the controls due to differences in head size, slight modifications in the parameters and resolutions were observed. Consequently, 16 genetically verified EPM1 patients (10 male, mean age of 31.0±10.9 years, range 18−51 years) and 16 healthy controls (8 men, mean age of 35.2±12.0 years, range 19−52 years) shared identical magnetization-prepared rapid acquisition of gradient echo sequence details and were included in the 3D TA study.

### Clinical Assessment of Patients with EPM1

Of the EPM1 patients, 13 were homozygous for the dodecamer expansion mutation, while 3 were compound heterozygous for the expansion mutations. The mean onset age was 9.6±1.9 years (range 5−12 years) and the mean duration of the disorder at the time of the study was 21.4±10.2 years (range 8−41 years). All of the EPM1 patients were treated with antiepileptic drugs (AEDs). Valproate was in use in all 16 patients and was augmented with levetiracetam (n = 10), clonazepam (n = 10), topimarate (n = 3) piracetam (n = 4), lamotrigine (n = 5), clopazam (n = 1) or other AEDs (n = 5). The medical histories of EPM1 patients were confirmed from medical records and by interviewing the patients and their relatives. A Unified Myoclonus Rating Scale (UMRS) test panel was performed as part of the clinical patient evaluation. UMRS is a quantitative, 74-item clinical rating instrument comprising 8 sections [34]. The patients were video-recorded and evaluated by using the standard protocol. Higher UMRS scores indicate more severe myoclonus.

Neuropsychological assessments were performed by an experienced neuropsychologist (M.Ä). General intellectual ability was assessed with the Wechsler Adult Intelligence Scale Revised (WAIS-R) [35], and verbal and performance Intelligence Quotients (VIQ, PIQ) were estimated.

### MR Image Acquisition

The EPM1 patients and healthy control subjects underwent MRI of the brain (1.5 T, Siemens Magnetom Avanto, Erlangen, Germany) using a birdcage Tx/Rx head coil. T1-weighted 3D images (magnetization-prepared rapid acquisition of gradient echo: TR 1980 ms, TE 3.93 ms, flip angle 15°, matrix 256×256, 176 sagittal slices, slice thickness 1.0 mm, in-slice resolution of 1.0 mm×1.0 mm) were used for regional 3D TA.

### Texture Analysis and Volumes of Interest Definition

TA was performed with the software package MaZda (MaZda 4.60 3D, Institute of Electronics, Technical University of Lodz, Poland) [36], [37]specially designed for texture analysis by Materka and co-workers as a part of the European COST B11 and the following COST B21 programs.

**Figure 1:**
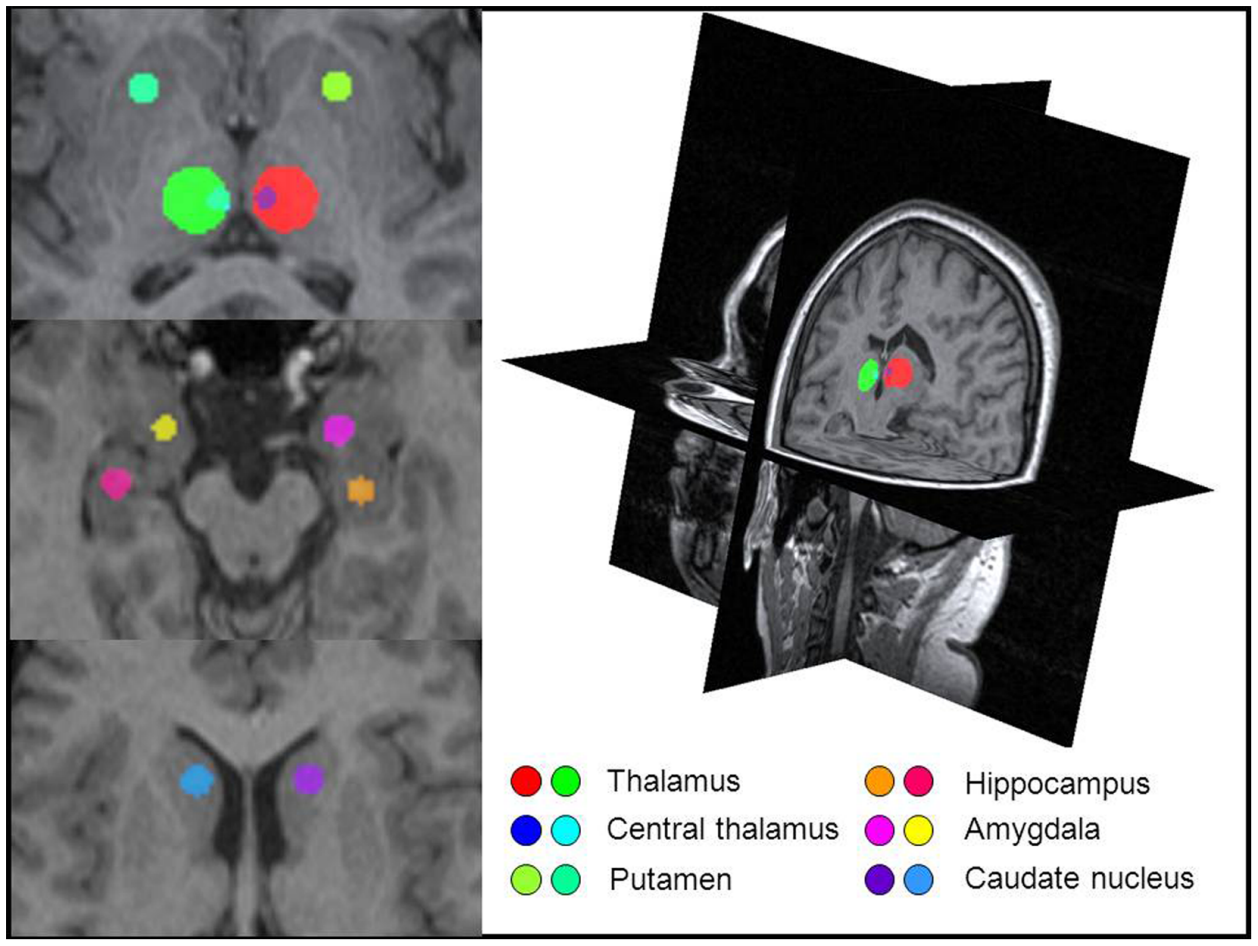
Volumes of interests and a three-dimensional view of the brain in MaZda. Image slices from a T1-weighted 3D image package illustrating the volumes of interest in a 19 year old female patient. There is no focal pathology or atrophy visible. VOIs are placed bilaterally in the thalamus, hippocampi, amygdalae, caudate nuclei and putamen.

Spherical volumes of interest (VOI) were manually placed bilaterally on each region of interest in the deep gray matter structures (thalamus, putamen, caudate nucleus, hippocampus and amygdala; Figure 1). The VOIs were carefully placed to avoid any overlap with other anatomical structures or cerebrospinal fluid. The 3D VOI placement was done manually by two observers (S.S. and K.H.).

Image gray level intensity normalization was performed with method limiting image intensities in the range [μ−3σ, μ+3σ], where μ is the mean gray level value and σ the standard deviation. This method has been shown to intensify differences between two classes when comparing image intensity normalization methods in texture classification [38].

A total of 223 texture parameters were calculated based on the histogram, gradient, run-length matrix and co-occurrence matrix (Table 1) [36], [37]. Run-length matrix parameters were calculated in four directions: horizontal (0°), vertical (90°), 45° and 135°, and co-occurrence matrix parameters were calculated in three distances of 1, 2 and 3 voxels in each 3D spatial co-ordinate directions were considered. All of these texture features were calculated for each VOI.

**Table 1:** Texture features used in the study.

Histogram	Absolute gradient	Co-occurrence matrix	Run-length matrix
Mean	Mean	Angular second moment	Run-length non-uniformity
Variance	Variance	Contrast	Gray-level non-uniformity
Skewness	Skewness	Correlation	Long run emphasis
Kurtosis	Kurtosis	Sum of squares	Short run emphasis
Percentiles 1-, 10-, 50-, 90-, 99-%	Percentage of pixels with non-zero gradient	Inverse difference moment	Fraction image in runs
		Sum average	
		Sum variance	
		Sum entropy	
		Difference variance	
		Difference entropy	

### Statistical Analysis

Statistical analyses were performed with SPSS 19.0 (IBM SPSS, Chicago, Illinois). P-values under 0.05 were considered statistically significant. Because of the small group size and skewed distributions, nonparametric statistical tests were used. The Mann-Whitney U test was used to evaluate the raw TA parameters to describe the textural difference between EPM1 patients and controls on each VOI. All 223 raw texture parameters were statistically tested to find out how many and which of the 223 parameters differed statistically.

The texture parameters were calculated in several directions and pixel distances, mean value for different pixel distances, and directions were calculated for six texture parameters (Table 2) and for correlation analysis of four texture parameters (entropy, angular second moment, short run emphasis, long run emphasis). Thus, the Spearman correlation coefficient was used to assess any correlations between the mean values of the four texture parameters and the clinical parameters (myoclonus in action score, age, duration of the disease, PIQ and VIQ).

**Table 2:** Texture features that differed most between the patients with EPM1 and healthy controls.

	Patient		Control		
	Mean	SD	Mean	SD	p
**Histogram-based parameters**					
**Mean 3D**					
VOI1, right thalamus	677.71	24.03	664.88	22.06	0.152
VOI2, left thalamus	682.10	23.13	658.23	21.71	0.004
VOI9, right putamen	631.92	21.25	653.66	24.70	0.016
**Variance 3D**					
VOI1, right thalamus	1571.64	353.99	1148.05	204.36	0.001
VOI2, left thalamus	1500.03	215.39	1113.45	160.68	0.000
VOI9, right putamen	682.85	226.09	542.93	127.31	0.050
**Co-occurence-based parameters**					
**Mean of angular second moment**					
VOI1, right thalamus	5.55×10^−4^	1.42×10^−5^	5.76×10^−4^	1.52×10^−5^	0.001
VOI2, left thalamus	5.57×10^−4^	1.05×10^−5^	5.79×10^−4^	1.21×10^−5^	0.000
VOI9, right putamen	9.06×10^−3^	2.96×10^−4^	9.38×10^−3^	3.14×10^−4^	0.006
**Mean of entropy**					
VOI1, right thalamus	3.28	8.50×10^−3^	3.27	8.61×10^−3^	0.001
VOI2, left thalamus	3.28	6.03×10^−3^	3.27	6.93×10^−3^	0.000
VOI9, right putamen	2.11	1.04×10^−2^	2.09	1.24×10^−2^	0.005
**Run-length matrix-based parameters**					
**Mean of short run emphasis**					
VOI1, right thalamus	9.91×10^−1^	7.84×10^−4^	9.90×10^−1^	1.14×10^−3^	0.000
VOI2, left thalamus	9.91×10^−1^	1.53×10^−3^	9.90×10^−1^	1.42×10^−3^	0.042
VOI9, right putamen	9.93×10^−1^	2.46×10^−3^	9.91×10^−1^	3.30×10^−3^	0.101
**Mean of long run emphasis**					
VOI1, right thalamus	1.035	3.30×10^−3^	1.042	4.63×10^−3^	0.000
VOI2, left thalamus	1.038	6.18×10^−3^	1.043	5.72×10^−3^	0.026
VOI9, right putamen	1.029	1.02×10^−2^	1.037	1.38×10^−2^	0.109
**Run-length non-uniformity**					
VOI1, right thalamus	1381.49	7.98	1372.74	13.29	0.046
VOI2, left thalamus	1377.92	10.98	1369.90	17.78	0.050
VOI9, right putamen	140.79	2.49	137.49	3.45	0.002
**Gray-level non-uniformity**					
VOI1, right thalamus	11.41	1.00	12.95	1.21	0.001
VOI2, left thalamus	11.48	0.70	13.14	0.99	0.000
VOI9, right putamen	2.58	0.34	2.74	0.18	0.032

Mean 3D and Variance 3D are single texture features whereas co-occurrence and run-length matrix-based parameters are the mean of each texture feature.

To test the reproducibility of the TA, 10 control subjects were drawn by two observers. The intraclass correlation coefficient (ICC) with a 95% confidence interval, coefficient of variation (CV) and paired samples t-test were calculated. Co-occurrence parameters from one voxel distance (1, 0, 0) i.e., all together 1320 numerical values per observer were involved in the reproducibility analysis.

## Results

**Figure 2:**
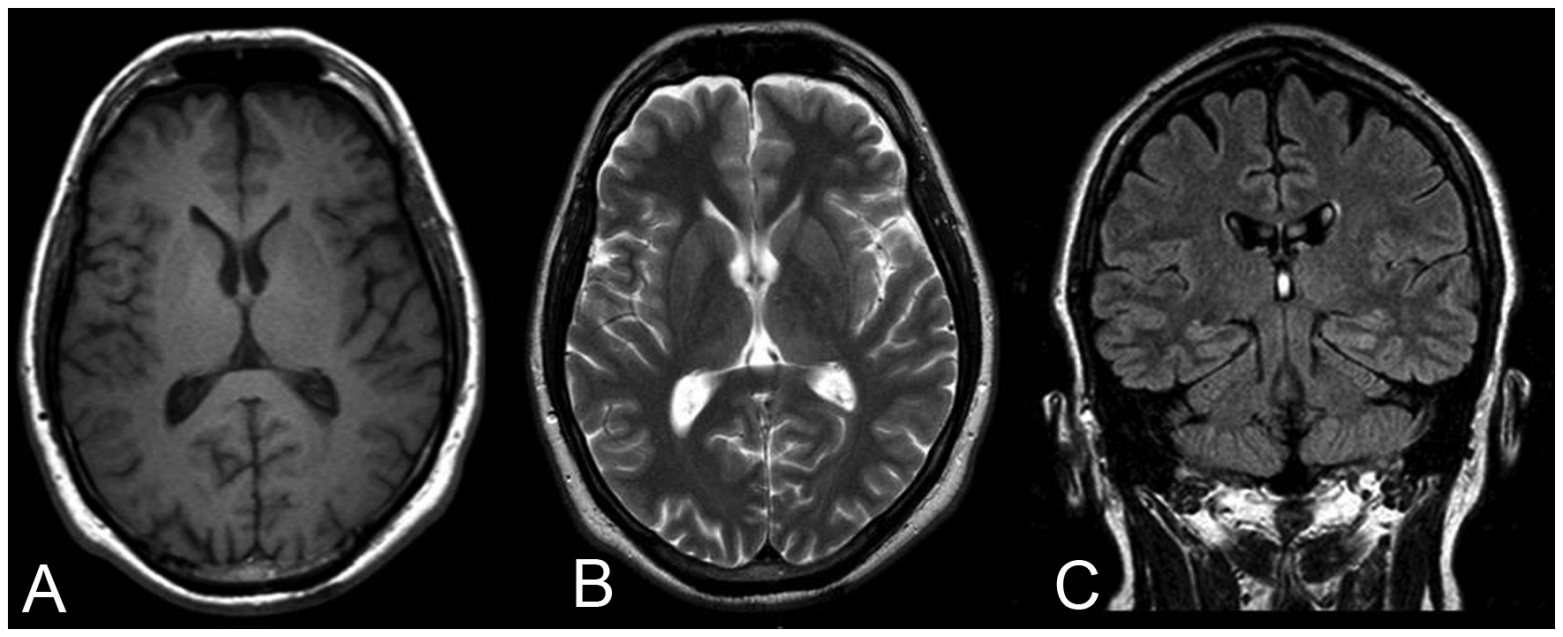
Illustrative images from a 34 year old male patient with EPM1. A) T1-weighted, B) T2-weighted and C) Fluid-Attenuated Inversion Recovery (FLAIR) MR images. Mild frontoparietal cortical atrophy can be suspected but there are no visible focal abnormalities.

The demographic and clinical data of patients with EPM1 are presented in Table 3. When assessed visually by experienced neuroradiologists (P.K. and R.V.), no focal signal intensity abnormalities were found (Figures 1 and 2).

**Table 3:** Demographic data of 16 EPM1 patients.

	n	Mean ± SD	Range
Sex, M/F	10/6		
Age, y		31.0±10.9	18−51
Age at EPM1 onset, y		9.6±1.9	5−12
Duration of disease, y		21.4±10.2	8−41
UMRS: Myoclonus with Action		49.1±25.8	12−92
Wheelchair use, no/occasionally/wheelchair bound	9/4/3		
Number of AED’s in use, 2/3/4/5	5/5/5/1		
Phenytoin use, never/earlier temporarily/unknown	9/6/1		
VIQ		82.4±12.4	62−102
PIQ		70.4±13.1	52−94

### Reproducibility

Reproducibility of 3D TA proved to be excellent. There were no statistically significant differences (p = 0.738) between Observer 1 and Observer 2 in the values of co-occurrence (1, 0, 0) parameters. ICC was excellent (0.990). The CV was 1.90 for Observer 1, and 1.89 for Observer 2.

### Regional Texture Parameters differing between Patients and Control Subjects

The regional differences in texture parameters (n = 223) were evaluated by the number of TA parameters with statistically significant differences between EPM1 patients and healthy controls (Table 4). The largest number of significant differences between EPM1 patients and healthy controls were found in the VOIs of the thalamus (Table 4) and were based on co-occurrence matrix, in particular the following parameters: angular second moment, entropy, sum variance and sum average (Table 5). The values of entropy were higher in patients than in healthy controls whereas the angular second moment feature acted in the opposite direction (Table 2).

**Table 4:** Volumes of interest (VOI) with number and percentages of statistically significantly different texture parameters from a total of 223 texture parameters between EPM1 patients and healthy controls.

Region	Volume of VOI (mm^3^)	Number of statistically significant different parameters	%
VOI 1, Right side of the thalamus	1437	62	28%
VOI 2, Left side of the thalamus	1437	83	37%
VOI 3, Right central thalamus	66	27	12%
VOI 4, Left central thalamus	66	12	5%
VOI 5, Right amygdala	144	15	7%
VOI 6, Left amygdala	144	11	5%
VOI 7, Right hippocampus	144	4	0.5%
VOI 8, Left hippocampus	144	11	5%
VOI 9, Right putamen	144	59	26%
VOI 10, Left putamen	144	10	1%
VOI 11, Right caudate nucleus	144	7	3%
VOI 12, Left caudate nucleus	144	16	7%

**Table 5:** The number of differing co-occurence based texture parameters (p<0.05) between EPM1 patients and healthy controls in the thalamus and putamen in three distances in all five evaluated directions (maximal number = 15 parameters).

	Thalamus (right side)	Thalamus (left side)	Putamen right	Putamen left
Angular second moment	**14/15**	**15/15**	6/15	1/15
Entropy	**14/15**	**15/15**	**9/15**	0/15
Sum average	**7/15**	1/15	2/15	0/15
Sum variance	1/15	**8/15**	0/15	0/15
Sum entropy	3/15	3/15	5/15	0/15
Sum of squares	4/15	0/15	4/15	1/15
Contrast	1/15	6/15	1/15	1/15
Correlation	1/15	6/15	0/15	0/15

The most different texture parameters are displayed in bold.

The thalamus also differed in the histogram-based features including variance and mean as EPM1 patients had higher values than healthy controls. Furthermore, the run-length matrix-based parameters also differed between patients and controls (Table 6). The values of short run emphasis were larger in patients than in healthy controls, whilst values of long run emphasis features acted in the opposite direction (Table 2). Values indicating gray-level non-uniformity were smaller in patients than in controls and vice versa with run-length non-uniformity (Table 2).

**Table 6:** The directions of differing run-length-based texture parameters (p<0.05) between EPM1 patients and healthy controls in the thalamus and putamen.

	Thalamus (right side)	Thalamus (left side)	Putamen right	Putamen left
Long run emphasis	Horizontal	Horizontal	135°	45°
	135°	Vertical		
Short run emphasis	Horizontal	Vertical	135°	45°
	135°			
Run-length non-uniformity	135°	Horizontal	Horizontal	–
		Vertical	45°	
			135°	
Gray-level non-uniformity	All degrees	All degrees	All degrees	–

In addition to the thalamus bilaterally, the right putamen provided statistically differing texture features between EPM1 patients and healthy controls (Tables 4 and 2). Again, significant differences were found in histogram-, co-occurrence matrix- and run-length matrix-based parameters. No major differences were found between EPM1 patients and healthy controls in the left putamen, hippocampi, amygdalae or caudate nuclei.

### Correlations between TA and Clinical Parameters

The myoclonus in action score correlated significantly with the angular second moment values in the left side of the thalamus (VOI2, r = 0.542, p = 0.030) and tended to correlate with values from the right side of the thalamus (VOI1, r = 0,440, p = 0.088). A tendency for inverse correlation was observed between the myoclonus in action score and the entropy values both in the right side of the thalamus (r = −0.442, p = 0.087) and left side of the thalamus (r = −0.495, p = 0.051). No correlations were found between the texture parameters in the thalamus and age, duration of the disorder, PIQ and VIQ. In the right putamen, the VIQ score significantly correlated with the entropy values (r = −0.498, p = 0.050) and angular second moment values (r = 0.514, p = 0.042). No correlations were found between the texture parameters in the right putamen and myoclonus in action score, age, duration of the disorder or PIQ.

## Discussion

The present study shows that MRI-based texture analysis reveals imperceptible alterations in EPM1 patients, especially in the thalamus. Three dimensional TA is a novel method for the analysis of MR images of the brain, and according to our results, 3D TA is able to provide subtle information of the structures of deep gray matter in EPM1 patients that could not be detected by direct visual inspection of the images. The TA changes were demonstrated by statistical analysis of the pixel gray level distributions in the volumes of interest in the anatomical structures analysed.

In our study we used 3D texture analysis, a more advanced technique than reported in most of the previous TA studies [17]. Studies of TA in epilepsy are sparse but have evaluated hippocampal abnormalities in temporal lobe epilepsy [21], [24], [26], [27] and observed subtle lesions of cortex in focal cortical dysplasia [22], [23]. Recently, 2D texture analysis based on co-occurrence matrix was able to detect tissue alterations in the right side of the thalamus in JME, and both caudate nuclei and the thalamus in Machado-Joseph disease, a rare neurodegenerative disorder characterized by ataxia and motorical dysfunction [25], [39].

The present TA study detected significantly different thalamic texture features in EPM1 patients compared to healthy controls. This is in line with imaging and experimental studies that have previously suggested thalamic and possible dopaminergic pathology in EPM1 [11], [13], [15], [40]–[42]. The thalamus is a relay centre between subcortical areas and the cerebral cortex, and it has multiple sensory and motor functions, along with the regulation of awareness, attention, memory and language [43]–[45]. Lesions confined to the thalamus have been associated with asterixis [46], and hemorrhages restricted to the region lateralis of the thalamus lead to a cheiro-oral syndrome [47] or choreiform and dystonic movements associated with myorhythmia [48]. Lesions of both thalamus and basal ganglia are related to dystonia [49]. Similar clinical symptoms are seen in both EPM1 patients [6] and *Cstb*-deficient mice [50] in the shape of ataxia, apraxia, dysarthia and involuntary asynchronous myoclonic jerks that occur mainly in the proximal muscles of the extremities.

The most significantly different parameters in the thalami of EPM1 patients compared to controls were the second-order co-occurrence matrix-based parameters, especially angular second moment and entropy values. Angular second moment is a measure of homogeneity as it measures the monotony of gray level transition in an image texture. The observed higher value of this feature in control individuals indicates that the intensity varies less in the VOI and the VOI is more homogenous than in EPM1 patients. Entropy indicates the complexity and randomness within the VOI. When the image is not texturally uniform then the value of entropy is larger [18]. In other words, our results indicate that the thalamus may be structurally more complex and heterogenous in EPM1.

We also observed differences in first-order histogram-based parameters and higher-order RLM-based parameters. Histogram-based statistics assess the global distribution of pixels/voxels with specific gray level tones [17]. Several statistical properties can be calculated from the histogram. The mean is the average intensity level of the image, variance assesses the roughness of an image, skewness describes the histogram symmetry and kurtosis describes the flatness of the histogram. We found that EPM1 patients had higher values of variance in the thalamus than those of the control group, thus the thalamic texture in EPM1 patients is rougher than that of healthy controls.

RLM-based parameters assess the number of runs when two or more pixels/voxels have the same value in a present direction, and they describe the coarseness or smoothness of an image [51]. Gray-level non-uniformity calculates how uniformly the runs are distributed among the gray levels; smaller values indicate that the distribution of runs is more uniform. The value of short run emphasis is larger in more coarse images and the value of long run emphasis is larger in smoother images. In the present study, the short run emphasis features were larger in the thalami of EPM1 patients than in healthy controls, indicating a more coarse thalamic texture.

Put together, the observed differences in variance, angular second moment, entropy and short run emphasis features indicate that the texture of the thalamus may be more complex, rough and coarse in EPM1 patients than in healthy controls.

The putamen together with the caudate nucleus and nucleus accumbens form the striatum, which is a major part of the basal ganglia. Basal ganglia are a part of the extrapyramidal motor system involved in cognition, emotion and motivation [52]. The anterior parts of the striatum, including the putamen, are essential for learning, attention, planning [53], [54] and verbal working memory [55], where dopamine plays an essential role in neurotransmission [56]. Putamenal pathology is related to movement disorders that also contain cognitive features, for example Parkinson’s disease and Huntington’s disease [57]. Interestingly, microstructural and volumetric abnormalities of the putamen have also been observed in juvenile myoclonic epilepsy [58] which shares similar clinical characteristics with EPM1.

Our results indicated that the right putamen had significantly different texture parameters in EPM1 patients when compared to the healthy controls. The most significantly different parameters were the same as those found in the thalamus bilaterally; co-occurrence matrix based angular second moment and entropy. The VIQ correlated negatively with entropy and positively with angular second moment values in the right putamen, indicating that the smaller the VIQ score, the more complex the texture in the right putamen. There was no correlation between myoclonus in action scores and entropy in the putamen of either hemisphere. Our VOI included the anterior, mainly rostrodorsal (associative) part of the putamen that is involved in learning new motor tasks, whereas the posterior caudoventral (sensorimotor) part of putamen is more involved in storage and execution of learned tasks [59], [60]. The parallel textural alterations in the thalamus and right putamen are an interesting finding. Initially, basal ganglia-thalamocortical circuitry was considered to be involved only in the control of movement but nowadays these structures are also regarded to be part of higher-order behavioral control [52], [63].

Memory problems are not main symptoms in EPM1 [61], [62] and previous imaging studies have not revealed any hippocampal abnormalities. In agreement with the previous studies, we found no major textural differences in the hippocampi, amygdalae or caudate nuclei between the EPM1 patients and healthy controls.

Our study has some limitations. The original study population had in part been scanned with slightly different MR imaging parameters. Since we wanted to make sure the different imaging parameters do not affect the TA results, our identical MRI data for TA remained relatively small. Further, some of the original patient imaging data could not be included in the texture analysis because of the suboptimal image quality caused by myoclonic movement artefacts. Finally, 16 patients and 16 controls from the original study population shared the identical imaging protocol and were included in the present TA study. The restrictions in the data collection reflect the fact that TA is still an experimental method with many limitations, including its potential sensitivity to slight differences in different scanners, coils and imaging protocols, complicating its routine clinical use and prohibiting data comparisons between institutions.

To conclude, three dimensional TA proved to be a feasible method to obtain imperceptible quantitative individual information from MR images of the brain in EPM1. Patients with EPM1 exhibit more coarse and heterogeneous texture in thalamus and right putamen. The textural differences observed in the present study parallel the previous imaging, neuropathological, and molecular genetics studies of thalamic involvement in EPM1. Textural alterations in the right putamen is a novel finding. Our results indicate that the changes in both the thalamus and putamen may play an important role in the pathophysiology of EPM1. Further studies in larger patient materials will show whether 3D TA could be a relevant tool for clinical applications.
